# Antibodies against type I interferon: detection and association with severe clinical outcome in COVID‐19 patients

**DOI:** 10.1002/cti2.1327

**Published:** 2021-08-17

**Authors:** David Goncalves, Mehdi Mezidi, Paul Bastard, Magali Perret, Kahina Saker, Nicole Fabien, Rémi Pescarmona, Christine Lombard, Thierry Walzer, Jean‐Laurent Casanova, Alexandre Belot, Jean‐Christophe Richard, Sophie Trouillet‐Assant

**Affiliations:** ^1^ Immunology Department Lyon Sud Hospital Hospices Civils de Lyon Pierre‐Bénite France; ^2^ CREATIS CNRS UMR5220 Inserm U1044 INSA Lyon University Lyon France; ^3^ Intensive Care Unit Hospices Civils de Lyon Lyon France; ^4^ Laboratory of Human Genetics of Infectious Diseases Necker Branch INSERM U1163 Necker Hospital for Sick Children Paris France; ^5^ Imagine Institute University of Paris Paris France; ^6^ St. Giles Laboratory of Human Genetics of Infectious Diseases Rockefeller Branch The Rockefeller University New York NY USA; ^7^ International Center of Research in Infectiology INSERM U1111 CNRS UMR 5308 ENS UCBL Lyon University Lyon France; ^8^ Infective Agents Institute Hospices Civils de Lyon Lyon France; ^9^ National Referee Centre for Rheumatic and AutoImmune and Systemic diseases in childrEn (RAISE) Lyon France; ^10^ Pediatric Nephrology, Rheumatology, Dermatology Unit Hospices Civils de Lyon Lyon France

**Keywords:** autoantibodies, COVID‐19, intensive care unit, SARS‐CoV‐2 virus, type I interferon, viral infection

## Abstract

**Objectives:**

Impairment of type I interferon (IFN‐I) immunity has been reported in critically ill COVID‐19 patients. This defect can be explained in a subset of patients by the presence of circulating autoantibodies (auto‐Abs) against IFN‐I. We set out to improve the detection and the quantification of IFN‐I auto‐Abs in a cohort of critically ill COVID‐19 patients, in order to better evaluate the prevalence of these Abs as the pandemic progresses, and how they correlate with the clinical course of the disease.

**Methods:**

The concentration of anti**‐**IFN‐α_2_ Abs was determined in the serum of 84 critically ill COVID‐19 patients who were admitted to ICU in *Hospices Civils de Lyon*, France, using a commercially available kit (Thermo Fisher, Catalog #BMS217).

**Results:**

A total of 21 of 84 (25%) critically ill COVID‐19 patients had circulating anti‐IFN‐α_2_ Abs above cut‐off (> 34 ng mL^−1^). Among them, 15 of 21 had Abs with neutralising activity against IFN‐α_2_, that is 15 of 84 (18%) critically ill patients. In addition, we noticed an impairment of the IFN‐I response in the majority of patients with neutralising anti‐IFN‐α_2_ Abs. There was no significant difference in the clinical characteristics or outcome of with or without neutralising anti‐IFN‐α_2_ auto‐Abs. We detected anti‐IFN‐α_2_ auto‐Abs in COVID‐19 patients' sera throughout their ICU stay. Finally, we also found auto‐Abs against multiple subtypes of IFN‐I including IFN‐ω.

**Conclusions:**

We reported that 18% of critically ill COVID‐19 patients were positive for IFN‐I auto‐Abs, whereas all mild COVID‐19 patients were negative, confirming that the presence of these antibodies is associated with a higher risk of developing a critical COVID‐19 form.

## Graphical abstract

We report here that 18% of severe COVID‐19 patients were positive for autoantibodies able to neutralize type I interferon (IFN). This finding further confirms the deleterious role of IFN‐I auto‐Abs in the antiviral immune response supporting the use of recombinant type I IFNs not targeted by the auto‐Abs (e.g. IFN‐β) in COVID‐19 patients with an impairment of the IFN‐I response.

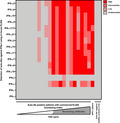

## Introduction

Severe acute respiratory syndrome coronavirus‐2 (SARS‐CoV‐2) infection leads to coronavirus disease 19 (COVID‐19), whose spectrum of clinical presentations is wide and includes severe pneumonia. The anti‐SARS‐CoV‐2 immune response has been extensively studied, and defects in antiviral mechanisms have been linked to disease severity. In particular, impairment of type I interferon (IFN‐I) immunity has been reported in critically ill COVID‐19 patients. Such defect can be because of either inherited genetic deficiencies in the IFN‐I pathway or the occurrence of circulating autoantibodies (auto‐Abs) directed against 14 or the 17 individual IFN‐I.^1^, ^2^, ^3^, ^4^ These auto‐Abs have also been detected in a third of patients from a small international cohort who had suffered from severe adverse events following yellow fever vaccination (YFV‐17D).^5^ These findings advocate for the development of diagnostic tools for the detection of IFN‐I auto‐Abs in routine laboratories, in order to identify early patients at risk of developing severe forms of COVID‐19 and to analyse the prevalence of IFN‐I Abs as the pandemic progresses and the virus evolves. To this aim, we tested a commercially available kit measuring IFN‐I auto‐Abs levels in the serum of COVID‐19 patients.

## Results

A total of 84 critically ill COVID‐19 patients, 11 patients with autoimmune polyendocrinopathy type 1 syndrome (APS‐1), 10 mildly symptomatic COVID‐19 healthcare workers and 76 healthy controls were included in the study. The critically ill COVID‐19 patients were admitted to ICU in the Lyon University Hospital, France, between September and December 2020. The presence of anti‐IFN‐α_2_ Abs was investigated: we first sought to determine a positive cut‐off value for Abs detection by performing measurements in 76 putative control sera, that is from healthy donors retrieved before the COVID‐19 outbreak. The mean value +3 standard deviation of these measurements provided a cut‐off value at 34 ng mL^−1^. We then assessed the presence of IFN‐α_2_ Abs in putative positive sera, that is sera from patients with autoimmune polyendocrinopathy type 1 syndrome (APS‐1), a condition known to be associated with anti‐cytokine auto‐Abs. All APS‐1 patients tested (*n* = 11) had high titres of circulating anti‐IFN‐α_2_ auto‐Abs (> 100 ng mL^−1^). We also evaluated the presence of anti‐IFN‐α_2_ auto‐Abs in 10 mildly symptomatic COVID‐19 healthcare workers, and none of them was found positive.

**Figure 1:**
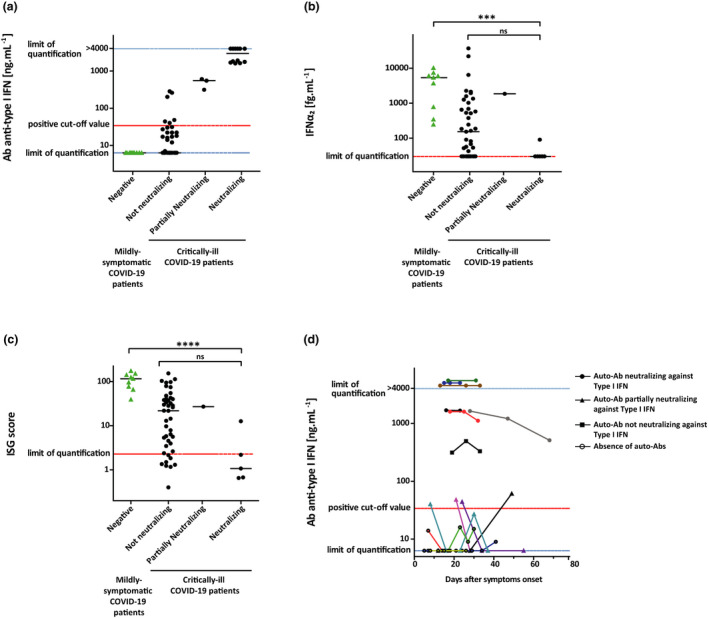
Anti‐type I IFN antibodies (Abs) in patients with life‐threatening COVID‐19. **(a)** Auto‐Abs concentrations with neutralizing capacity against IFN‐α. Concentration of auto‐Abs against IFN‐α_2_ (ng mL^−1^) was determined by a Thermo Fisher Kit (Catalog # BMS217) in serum samples collected from COVID‐19 patients admitted in ICU (*n* = 84) and COVID‐19 patients with mild respiratory symptoms (*n* = 10). **(b, c)** IFN‐α_2_ concentration (fg mL^−1^) **(b)** and ISG score **(c)** in plasma and whole blood collected from COVID‐19 patients in the first 15 days after symptom onset [critically ill COVID‐19 patients (*n* = 54) and mildly symptomatic COVID‐19 patients (*n* = 10)]. **(d)** Longitudinal detection of auto‐Abs against IFN‐α_2_ in COVID‐19 patients' serum during their ICU stay according to the delay post‐symptom. Dotted lines represent positive cut‐off value (threshold), lower limit of quantification (LLOQ) and upper limit of quantification (ULOQ). Solid black lines represent median. Comparisons were performed using the Kruskal–Wallis test followed by Dunn's test. ***P‐value ≤ 0.001 and ****P‐value ≤ 0.0001.

**Figure 2:**
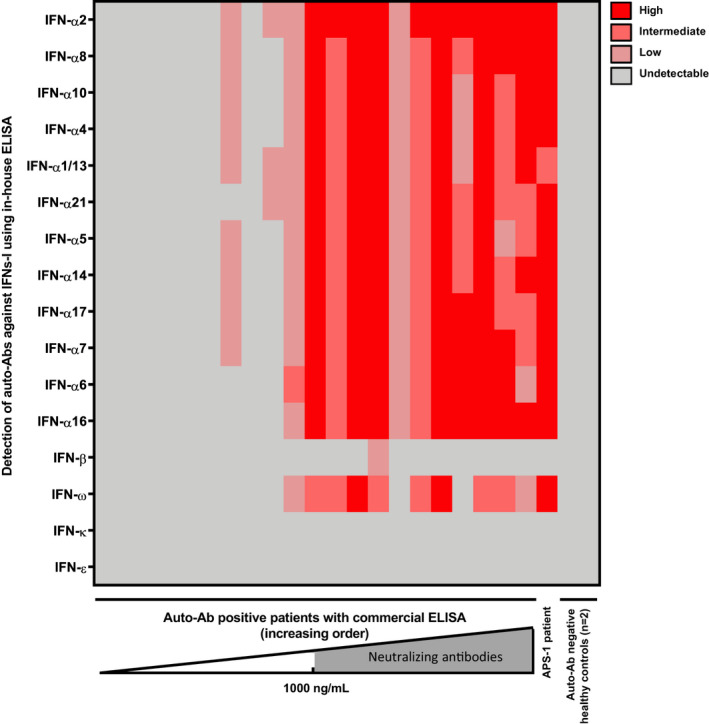
Auto‐Abs against other subtypes of IFN‐I. The presence of auto‐Abs against other subtypes of IFN‐I was assessed by in‐house ELISA in all sera with anti‐IFN‐α_2_ auto‐Abs detected with the Thermo Fisher Kit (*n* = 21, from left to right, increasing order of concentration of anti‐IFN‐α_2_ using the Thermo Fisher Kit). APS‐1 patient's serum was used as positive control, and sera from two healthy controls were used as negative controls.

We then measured anti‐IFN‐α_2_ Abs levels in the sera from the critically ill COVID‐19 patients: 21 of 84 (25%) were positive and had values above the cut‐off (> 34 ng mL^−1^). The neutralising capacity of their sera against IFN‐α was then evaluated as previously described.^5^ A neutralising activity was observed in 15 of 21 positive sera; in other words, 15 of 84 (18%) critically ill COVID‐19 patients had neutralising anti‐IFN‐α auto‐Abs (Figure 1a). Importantly, all sera with a titre of anti‐IFN‐α_2_ auto‐Abs above 1 µg mL^−1^ potently neutralised IFN‐α *in vitro*. In addition, in most patients with neutralising IFN‐I Abs, we noticed an impairment of the IFN‐I response, which was determined by the measurement of (1) plasma IFN‐α_2_ levels using the new digital ELISA technology single‐molecule arrays (Simoa) and (2) blood interferon stimulating gene (ISG) expression using the NanoString nCounter technology in blood samples collected in the first 15 days after symptom onset (Figure 1b and c). Moreover, there was no significant difference in the clinical characteristics (age, sex ratio and comorbidity) or outcome (death and O_2_ support) of critically ill COVID‐19 patients with or without neutralising anti‐IFN‐I auto‐Abs (Table 1). Then, serial measurement of IFN‐I auto‐Abs level during ICU stay was performed for seven positive patients. The level of IFN‐α_2_ auto‐Abs remained relatively stable across measurements performed up to 40 days apart (Figure 1d). Finally, we assessed the presence of auto‐Abs against other IFNs‐I in all sera positive for IFN‐α_2_ auto‐Abs (*n* = 21). Consistently with previous results,^1^ we observed that sera with anti‐IFN‐α_2_ auto‐Ab titres above 1 µg mL^−1^ also contained auto‐Abs against other subtypes of IFN‐α and 10 of 12 contained anti‐IFN‐ω auto‐Abs (Figure 2). Of note, these sera were able to neutralise IFN‐ω *in vitro*. Only one serum contained auto‐Abs targeting IFN‐β at a low titre, and none contained auto‐Abs against IFN‐ɛ or IFN‐κ.

**Table 1:** Clinical characteristics of critically ill COVID‐19 patients admitted in the intensive care unit

Clinical features	Auto‐nAb Negative (*n* = 69)	Auto‐nAb Positive (*n* = 15)	*P*‐value
Age (years)	67 [58–72]	65 [55–74]	0.75
Male sex	54 (78%)	13 (87%)	0.72
BMI (kg m^2^)	29 [26–34]	29 [25–32]	0.49
Autoimmune disease	7 (10%)	2 (13%)	0.66
Time between 1st symptoms and ICU admission (days)	9 [7–12]	10 [7–11]	0.80
Maximal ventilatory support			
Standard oxygen only	4 (6%)	0 (0%)	0.33
High‐flow oxygen only	19 (28%)	7 (47%)	
Invasive mechanical ventilation	46 (67%)	8 (53%)	
ARDS criteria	44 (64%)	8 (53%)	0.65
Worst PaO_2_/FiO_2_ day 1 in ICU (mmHg)	75 [62–103]	89 [62–116]	0.40
ECMO	15 (22%)	2 (13%)	0.72
SOFA day 1 in ICU	4 [3‐8]	3 [2‐5]	0.11
SAPS2 day 1 in ICU	40 [31‐47]	43 [38‐48]	0.41
Vasopressor requirement in ICU	45 (65%)	9 (60%)	0.77
Renal replacement therapy in ICU	27 (39%)	4 (27%)	0.56
ICU length of stay (days)	13 [7–35]	13 [6–24]	0.74
ICU mortality	29 (42%)	5 (33%)	0.58

ARDS, acute respiratory distress syndrome; BMI, body mass index; ECMO, extracorporeal membrane oxygenation; ICU, intensive care unit; nAb, neutralizing Auto‐Abs against IFN‐α; SAPS2, Simplified Acute Physiology Score II; SOFA, sequential organ failure assessment score.

Data are expressed as median [IQR] or count (percentage). The Mann–Whitney and Fisher tests were used for quantitative and qualitative variables, respectively.

## Discussion and Conclusions

A previous study has reported that IFN‐I auto‐Abs were present in 10.2% of life‐threatening COVID‐19 patients, undetectable in 663 individuals with asymptomatic or mild COVID‐19, and detected in only 0.33% of healthy individuals.^1^ Here, 18% of critically ill COVID‐19 patients were positive for IFN‐I auto‐Abs, whereas all mild‐COVID‐19 patients were negative. We noticed that only a part of auto‐Abs detected were able to neutralise IFN‐I in the conditions we used, which confirms previous studies in COVID‐19 patients and systemic lupus erythematosus subjects.^1^, ^6^ This finding further confirms the deleterious role of IFN‐I auto‐Abs in the antiviral immune response and the importance of the IFN‐I pathway in the defence against SARS‐CoV‐2 infection. Based on its antiviral properties, recombinant IFN‐I has been tested as therapy for severe COVID‐19, but the treatment showed little or no benefit.^7^, ^8^ Yet, the potential of such treatment may have been hindered by the presence of IFN‐I auto‐Abs in patient sera, and this question could therefore be revisited by determining the level of these Abs, for example using the ELISA method we used here. Moreover, patients could be treated with recombinant IFN‐I that is not targeted by auto‐Abs (e.g. IFN‐β).

Finally, the detection of anti‐IFN‐I auto‐Abs in COVID‐19 patients could be useful in routine to identify patients at risk of developing a severe form of the disease. The technique we described here is adequate for this purpose as it can rapidly provide quantitative measurements and has a cut‐off correlated with neutralisation assays (1 µg mL^−1^). However, the presence of auto‐Abs was not associated with poorer outcome in critically ill patients and does not explain all the severe forms of COVID‐19, other causes should therefore be sought (e.g. cytokine release syndrome and presence of other risk factors such as obesity and hypertension).

## Methods

### Participants

#### Critically ill COVID‐19 patients

Plasma samples and PAXgene^®^ tubes were collected from COVID‐19 patients hospitalised in the university hospital of Lyon (Hospices Civils de Lyon), France. Diagnosis of COVID‐19 was established in all patients by RT‐PCR.

All critically ill patients positive for SARS‐CoV‐2 virus, admitted to ICU (Croix‐Rousse Hospital, Hospices Civils de Lyon), were included in the MIR‐COVID study. This study was registered to the *Commission nationale de l'informatique et des libertés* (CNIL, French data protection agency) under the number 20‐097 and was approved by an ethics committee for biomedical research (*Comité de Protection des Personnes HCL*) under the number N°20‐41. In agreement with the General Data Protection Regulation (Regulation (EU) 2016/679 and Directive 95/46/EC) and the French data protection law (Law n°78‐17 on 06/01/1978 and Décret n°2019‐536 on 29/05/2019), we obtained consent from each patient or his or her next of kin.

#### Mildly symptomatic COVID‐19 patients

Plasma samples and PAXgene^®^ tubes were collected from symptomatic healthcare workers upon COVID‐19 diagnosis. Written informed consent was obtained from all participants. The study was approved by the national review board for biomedical research in April 2020 (Comité de Protection des Personnes Sud Méditerranée I, Marseille, France; ID RCB 2020‐A00932‐37). The study was registered on ClinicalTrials.gov (NCT04341142) where the eligibility, inclusion and exclusion criteria are previously described.^9^

#### Healthy controls

Prepandemic serum was selected from healthy controls who were recruited among donors to the Lyon blood transfusion centre (Etablissement Français du Sang, EFS). According to French procedures, a written nonopposition to the use of donated blood for research purposes was obtained from HCs. The donors' personal data were anonymised before transfer to our research laboratory. We obtained approval from the local ethical committee and the French Ministry of Research (DC‐2008‐64) for handling and conservation of these samples.

### Auto‐Abs anti‐IFN‐I

The presence of anti‐IFN‐α_2_ auto‐Abs was assessed in the plasma using a commercially available kit (Thermo Fisher; Catalog # BMS217). The positive cut‐off value for Ab detection was 34 ng mL^−1^. The presence of auto‐Abs against other IFNs‐I was assessed using an ELISA technique, as previously described.^1^ Briefly, 96‐well ELISA plates were coated with different cytokines [rhIFN‐α_2_ (Miltenyi Biotec, ref. number 130‐108‐984), rhIFN‐ω (Merck, ref. number SRP3061) or cytokines from PBL Assay Science (Catalog #11002‐1) or IFN‐β (Miltenyi Biotec, ref. number: 130‐107‐888)] and incubated overnight at 4°C. Plates were then washed (PBS 0.005% Tween), then incubated with 5% nonfat milk powder in the same buffer, washed again and again incubated with 1:50 dilutions of plasma from patients or controls for 2 h at room temperature (or with specific mAbs as positive controls). Horseradish peroxidase (HRP)‐conjugated Fc‐specific IgG fractions from polyclonal goat antiserum against human IgG, IgM or IgA (Nordic Immunological Laboratories) were added to a final concentration of 2 μg mL^−1^ after thorough washing. Plates were then incubated for 1 h at room temperature and washed. HRP substrate was added, and the optical density (OD) was measured.

The neutralisation capacity of antibodies against IFN‐α_2_ and IFN‐ω was determined as previously described.^5^ Briefly, HEK‐293T cells were transfected with a plasmid encoding the firefly luciferase under the control of human *ISRE* promoters. Cells were then cultured in Dulbecco's modified Eagle's medium (DMEM; Thermo Fisher Scientific) supplemented with 10% healthy control or patient serum/plasma, and were either left unstimulated or were stimulated with IFN‐α_2_, IFN‐ω or IFN‐β (10 ng mL^−1^) for 16 h at 37°C. Finally, luciferase level was measured using the Dual‐Glo reagent, according to the manufacturer's instructions (Promega).

### Plasma protein quantification

The concentration of plasmatic IFN‐α (fg mL^−1^) was measured using single‐molecule array (Simoa) using a commercial kit for IFN‐α_2_ quantification (Quanterix™, Lexington, MA, USA). The assay was based on a 3‐step protocol and an HD‐1 Analyzer (Quanterix).

### IFN score assessment

Total RNA was extracted from whole blood stored in PAXgene^®^ tubes (Kit PreAnalytix, Qiagen©, SW) and was quantified using a spectrophotometer (NanoDrop 2000, Thermo Scientific™, MA, USA). RNA integrity was then assessed using the Agilent RNA microarray (Agilent Technologies©, Santa Clara, CA, USA). The expression of six ISGs [*interferon alpha‐inducible protein 27 (IFI27), interferon‐induced protein 44 like (IFI44L), interferon‐induced protein with tetratricopeptide repeats 1 (IFIT1), ISG15 ubiquitin‐like modifier (ISG15), radical S‐adenosyl methionine domain containing 2 (RSAD2)* and *sialic acid binding Ig like lectin 1 (SIGLEC1)*] and three housekeeping genes [*actin beta (ACTB), hypoxanthine phosphoribosyltransferase 1 (HPRT1)* and *RNA polymerase II subunit A (POLR2A)*] was quantified at the transcript level using the NanoString technology (NanoString Technologies©, WA, USA). Data standardisation was performed using the geometric mean of internal control and housekeeping gene counts. The ISG score was calculated as previously described.^10^

## Conflict of interest

The authors declare no conflict of interest.

## Author contributions

**David Goncalves:** Conceptualization; Formal analysis; Writing‐original draft. **Mehdi Mezidi:** Formal analysis; Resources; Writing‐review & editing. **Paul Bastard:** Conceptualization; Investigation; Methodology; Writing‐review & editing. **Magali Perret:** Investigation; Visualization; Writing‐original draft. **Kahina Saker:** Data curation; Investigation; Methodology. **Nicole Fabien:** Methodology; Resources; Validation; Writing‐original draft. **Rémi Pescarmona:** Investigation; Resources; Validation; Visualization. **Christine Lombard:** Methodology; Project administration; Writing‐original draft. **Thierry Walzer:** Supervision; Visualization; Writing‐original draft. **Jean‐Laurent Casanova:** Conceptualization; Validation; Visualization; Writing‐original draft. **Alexandre Belot:** Conceptualization; Supervision; Validation; Writing‐original draft. **Jean‐Christophe Richard:** Funding acquisition; Project administration; Resources; Writing‐original draft. **Sophie Trouillet‐Assant:** Conceptualization; Data curation; Formal analysis; Funding acquisition; Investigation; Methodology; Writing‐original draft; Writing‐review & editing.
